# An ECM-Mimetic Hydrogel to Promote the Therapeutic Efficacy of Osteoblast-Derived Extracellular Vesicles for Bone Regeneration

**DOI:** 10.3389/fbioe.2022.829969

**Published:** 2022-03-30

**Authors:** Kenny Man, Mathieu Y. Brunet, Angelica S. Federici, David A. Hoey, Sophie C. Cox

**Affiliations:** ^1^ School of Chemical Engineering, University of Birmingham, Birmingham, United Kingdom; ^2^ Trinity Centre for Biomedical Engineering, Trinity Biomedical Sciences Institute, Trinity College Dublin, Dublin, Ireland; ^3^ Dept. of Mechanical, Manufacturing, and Biomedical Engineering, School of Engineering, Trinity College Dublin, Dublin, Ireland; ^4^ Advanced Materials and Bioengineering Research Centre, Trinity College Dublin and RCSI, Dublin, Ireland

**Keywords:** extracellular vesicle, bone, controlled release, hydrogel, tissue engineering, drug delivery

## Abstract

The use of extracellular vesicles (EVs) is emerging as a promising acellular approach for bone regeneration, overcoming translational hurdles associated with cell-based therapies. Despite their potential, EVs short half-life following systemic administration hinders their therapeutic efficacy. EVs have been reported to bind to extracellular matrix (ECM) proteins and play an essential role in matrix mineralisation. Chitosan and collagen type I are naturally-derived pro-osteogenic biomaterials, which have been demonstrated to control EV release kinetics. Therefore, this study aimed to develop an injectable ECM-mimetic hydrogel capable of controlling the release of osteoblast-derived EVs to promote bone repair. Pure chitosan hydrogels significantly enhanced compressive modulus (2.48-fold) and osteogenic differentiation (3.07-fold), whilst reducing gelation times (2.09-fold) and proliferation (2.7-fold) compared to pure collagen gels (*p* ≤ 0.001). EV release was strongly associated with collagen concentration (R^2^ > 0.94), where a significantly increased EV release profile was observed from chitosan containing gels using the CD63 ELISA (*p* ≤ 0.001). Hydrogel-released EVs enhanced human bone marrow stromal cells (hBMSCs) proliferation (1.12-fold), migration (2.55-fold), and mineralisation (3.25-fold) compared to untreated cells (*p* ≤ 0.001). Importantly, EV-functionalised chitosan-collagen composites significantly promoted hBMSCs extracellular matrix mineralisation when compared to the EV-free gels in a dose-dependent manner (*p* ≤ 0.001). Taken together, these findings demonstrate the development of a pro-osteogenic thermosensitive chitosan-collagen hydrogel capable of enhancing the therapeutic efficacy of osteoblast-derived EVs as a novel acellular tool for bone augmentation strategy.

## Graphical Abstract

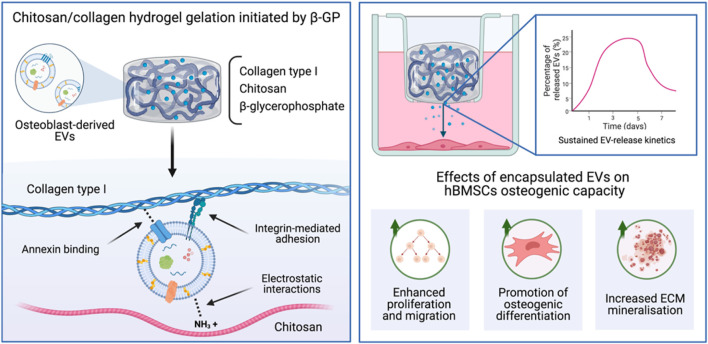

## 1 Introduction

There is a critical calling for therapeutic strategies to repair bone damage caused by traumatic injury, tumour resection, or other age-associated diseases such as osteoporosis (Baroli, 2009; Dimitriou et al., 2011). Approximately 10 million people suffer from musculoskeletal disorders in the United Kingdom, costing the National Health Service £5 billion annually (Chance-Larsen et al., 2019). Alarmingly, this is expected to escalate further in the future as a result of the growing ageing population and demand for continued quality of life, exacerbating the medical, and socio-economic burden worldwide. Traditional treatments such as autogenous or allogenous bone grafts have been seen as the gold standard for many decades, however, their use is associated with limitations such as donor site morbidity, limited availability and other complications (Betz, 2002; Roberts and Rosenbaum, 2012). Bone graft substitutes combined with hyper-concentrated osteoinductive growth factors have been utilised clinically with positive results (Roberts and Rosenbaum, 2012). For example, bone morphogenic protein 2 (BMP2) loaded collagen scaffolds (INFUSE^®^) has been widely used by orthopaedic surgeons to promote fracture repair (Gresham et al., 2021). However, these supraphysiological doses of BMP2 can result in severe complications including heterotopic ossification, hematoma, inflammation, and myelopathy that may require further surgical interventions (Epstein, 2013; Hustedt and Blizzard, 2014; James et al., 2016). Hence, there is a tremendous need for new approaches to rapidly regenerate bone, overcoming the limitations of current clinical strategies (Giannoudis et al., 2013).

Tissue engineering approaches for bone regeneration have been subject to extensive research in recent decades, with methods combining the use of cells with osteoinductive biomaterials as a promising strategy to promote osteogenesis. Cell-based therapies commonly utilise mesenchymal stromal cells (MSCs) due to their ease of procurement from numerous tissues and their multipotency (Marolt Presen et al., 2019; Man et al., 2021a). The use of these treatments are attractive as they attempt to mimic the body’s endogenous repair mechanisms, with some promising results observed (Tatara and Mikos, 2016). The direct transplantation of MSC-based therapies, however are associated with several complications including their uncontrolled differentiation, immunological rejection, their inherent heterogeneity, functional tissue engraftment, and neoplasm formation (Amariglio et al., 2009; Herberts et al., 2011). Moreover, the translation of cell-based therapies to the clinical setting is hindered by significant hurdles associated with intensive cost, government regulations, and ethical issues (Heathman et al., 2015). Consequently, there is great precedence to develop new treatments that retain the therapeutic effects of cell-based therapies.

In recent years, growing evidence has shown that bioactive factors secreted from cells play a critical role in the activation of stem/progenitor cell-mediated tissue repair (Kim et al., 2010; Li et al., 2016). Extracellular vesicles (EVs) are considered one of the most important secretory products of cells, involved in numerous trophic, and immunomodulatory processes. EVs are cell-secreted lipid nanoparticles that contain a diverse biological cargo including nucleic acids, proteins, and bioactive molecules (Raposo and Stoorvogel, 2013; Börger et al., 2017; Man et al., 2020). These nano-sized vesicles are thought to be heavily involved in intercellular communications, regulating tissue homeostasis and development (Yoon et al., 2014). The beneficial effects once attributed to cells are now thought to be partially due to the bioactive factors delivered by EVs (Gnecchi et al., 2005; Xin et al., 2014). Extensive research has investigated the use of EV-based therapeutics due to several advantageous properties when compared to cell-based treatments. For example, when compared to cells, EVs nanoscale size promotes administration, decreases vascular occlusion, and macrophage phagocytosis (van den Boorn et al., 2011; EL Andaloussi et al., 2013). Additionally, EVs possess high physiochemical stability and innate biocompatibility when compared to similarly sized delivery vehicles such as artificial nanoparticles (Clayton et al., 2003; Man et al., 2020). These naturally-derived nanoparticles are thought to be fundamentally involved in bone development, as matrix-bound EVs are critical for endochondral ossification (Ferreira and Porter, 2018; Ansari et al., 2021). Moreover, EVs derived from bone cells have been found to play a role in mediating intercellular communication, regulating bone homeostasis (Gao et al., 2018; Tao and Guo, 2019). Specifically, it has been shown that osteoblast-EVs contain RANKL, can stimulate RANKL-RANK signalling to promote osteoclast formation (Deng et al., 2015). In addition, RANK-containing osteoclast-EVs may obstruct RANKL-containing osteoblast-EVs function, thus inhibiting osteoclast formation (Huynh et al., 2016).Thus, harnessing these nanoparticles for regenerative medicine is an attractive acellular, but biological approach to recapitulate endogenous bone repair. Moreover, due to the limitations of current single growth factor treatments (e.g., BMP2) (Hustedt and Blizzard, 2014), the diverse biological cargo delivered by EVs presents a multitargeted strategy to stimulate tissue repair, an approach which is needed given the known synergistic role of angiogenesis, osteogenesis, innervation involved in bone regeneration (Grosso et al., 2017; Marrella et al., 2018). Previously, we reported that EVs derived from mineralising osteoblast were enriched with pro-mineralising proteins such as calcium channelling Annexins proteins, and several pro-osteogenic microRNA species (Davies et al., 2017; Man et al., 2021b). Hence, there have been intensive investigations into the role EVs may play as novel acellular tools that support natural bone regeneration, overcoming the tremendous regulatory hurdles associated with the translation of cell-based therapies (Sensebé et al., 2013; Heathman et al., 2015).

Several studies have demonstrated the osteoinductive capacity of EVs derived from stem/progenitor cells as an acellular approach to bone tissue engineering (BTE) (Davies et al., 2017; Yan et al., 2020; Man et al., 2021b; Man et al., 2021c). Although the potential of these nanoparticles have been shown, as with the administration of any bioactive molecules, controlling the half-life of EVs at the defect site is essential to therapeutic efficacy (Man et al., 2020). Previous studies have reported the rapid clearance of systemically administrated EVs, which accumulate in the liver and lungs and are sequestered by circulating macrophages in the reticuloendothelial systems and cleared from the body (Imai et al., 2015). Moreover, the direct administration of EVs into the site of injury has only transient effects as they are rapidly cleared from the defect site, often requiring subsequent injections to be clinically effective (Zhang et al., 2016). As such, there are growing investigations exploring the delivery of EVs within biomaterials to promote their bioavailability *in vivo*, ultimately improving their therapeutic efficacy (Brennan et al., 2020). The use of injectable polymeric biomaterials presents an attractive platform to tailor the release of these naturally-derived nanoparticles for different clinical requirements, in addition to allowing for minimally invasive delivery (Kearney and Mooney, 2013). For example, stem cell-derived EVs substantially improved cardiac function in mice following myocardial infarction when delivered in a gelatin methacryloyl (GelMA) hydrogel when compared to saline solution (Tang et al., 2021). Although several reports have demonstrated the sustained release kinetics of EVs from biomaterial systems (Mol et al., 2019; Nikravesh et al., 2019), there have been limited studies investigating the delivery of these nanoparticles within pro-osteoinductive materials to facilitate EV-induced bone formation. Hence, there is a current unmet clinical need to locally deliver these naturally-derived nanoparticles within biomaterials systems that control their release kinetics *in vivo*, in addition to promoting EV-induced bone formation.

Numerous natural and synthetic polymeric materials have been shown to exhibit pro-osteoinductive properties (Bai et al., 2018; Zhang et al., 2020). It has been reported that secreted EVs are sequestered by extracellular matrix (ECM) components (Huang et al., 2016; Man et al., 2020), therefore, the delivery of these nanoparticles within an ECM-mimetic biomaterial could provide a template for EV-induced mineralisation. Chitosan, a natural polysaccharide derived from deacetylated chitin, a structural component of crustacean exoskeletons (Saravanan et al., 2019), has been used for several applications due to its biocompatibility, antibacterial activity and low immunogenicity (Chung et al., 2012; Ahmadi et al., 2015). The polysaccharide unit of chitosan structurally resembles glycosaminoglycans (GAGs), a major component of the bone matrix (Khor and Lim, 2003). The cationic nature of chitosan is attributed to its biological properties such as antimicrobial activity, hemostasis and osteoconduction (Peschel et al., 2012). Additionally, the existence of hydroxyl and amino groups has been extensively exploited for the delivery of various growth factors or drugs (Zhang et al., 2010; Saravanan et al., 2019). Several studies have reported the enhanced therapeutic administration of EVs within thermosensitive chitosan hydrogels for numerous applications (Li et al., 2018; Shen et al., 2020; Zhao et al., 2021), harnessing the electrostatic interactions between cationic chitosan and the anionic EVs. To induced thermo-gelation within chitosan, the addition of β-glycerophosphate (β-GP) has commonly be employed to facilitate its sol-gel transition at physiological temperatures (Kong et al., 2018; Rahmanian-Devin et al., 2021). Moreover, it has been previously reported that EVs require a phosphate-rich environment to stimulate hBMSCs mineralisation (Davies et al., 2017). In BTE, chitosan has been combined with other materials to increase its osteoconductivity and mechanical strength (Di Martino et al., 2005). Type I collagen is the fundamental protein found in the bone matrix and has been widely utilised as a biomaterial since it supports cell adhesion, proliferation, and differentiation (Yang et al., 2004; Glowacki and Mizuno, 2008). Moreover, the integrin-mediated adhesion to collagen has been shown to enhance stem cell osteogenic differentiation (Salasznyk et al., 2004; Kundu and Putnam, 2006). This interaction has also been used to immobilise EVs within collagen hydrogels for different clinical applications (Buzás et al., 2018; Ramírez et al., 2020). Additionally, it has been reported that the calcium binding Annexin proteins found on the surface of mineralising vesicles are essential in mediating proteoliposome attachment to collagen fibrils (Kirsch et al., 2000; Davies et al., 2017). Hence, an ECM-mimetic composite hydrogel consisting of chitosan and type I collagen could control EV release kinetics and provide an osteoinductive delivery system to promote the therapeutic efficacy for EVs for bone repair.

**FIGURE 1:**
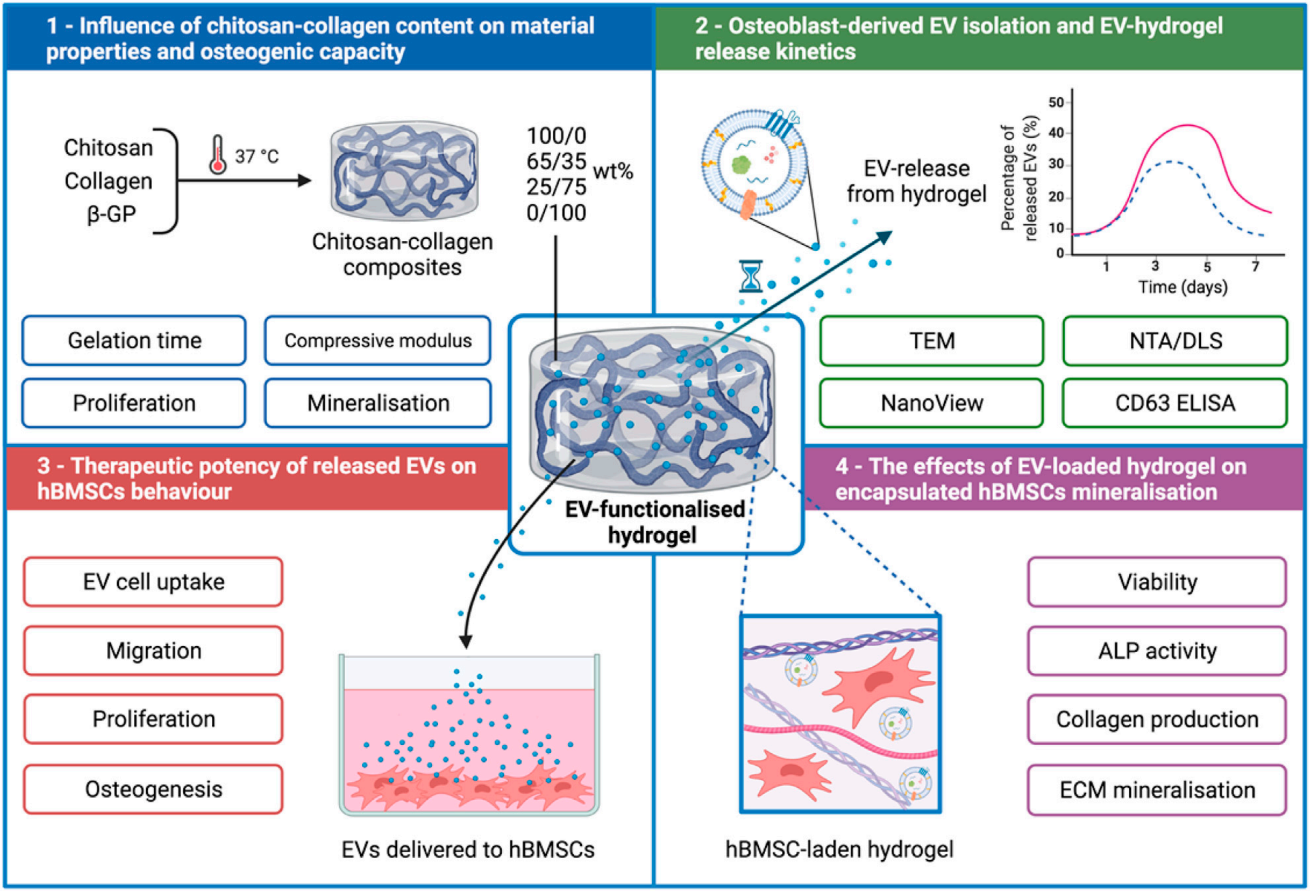
Experimental outline detailing the development of a chitosan-collagen composite hydrogel for promoting EV therapeutic efficacy for site specific bone regeneration. 1) The influence of chitosan-collagen hydrogel content on material properties and osteogenic differentiation. 2) EV isolation, characterisation, and hydrogel-EV release kinetics. 3) The biological efficacy of hydrogel-released EVs on hBMSCs behaviour. 4) The influence of EV-functionalised hydrogel on encapsulated hBMSCs mineralisation. Created with BioRender.com.

Therefore, in this present study, we developed an injectable thermo-responsive chitosan-collagen composite hydrogel with sustained EV release kinetics to promote bone formation. Initially, the influence of augmenting chitosan-collagen content on the hydrogel’s gelation time, mechanical strength and osteoinduction was investigated (Figure 1). EVs were isolated from mineralising osteoblasts and their release kinetics from these composite gels were analysed *via* a CD63 ELISA. The biological efficacy of hydrogel-released EVs on human bone marrow stromal cells (hBMSCs) proliferation and osteogenic differentiation was investigated. Finally, the effects of EV-functionalised hydrogels on encapsulated hBMSCs mineralisation was assessed.

## 2 Materials and Methods

### 2.1 Preparation of Chitosan-Collagen Hydrogels

Rat tail collagen type I (Corning, United Kingdom) was diluted in 0.02 M acetic acid to obtain a 4 mg/ml concentration. Medium molecular weight chitosan (Sigma-Aldrich, United Kingdom) was dissolved in 0.1 M acetic acid to obtain a 2 wt% chitosan stock solution. These two solutions were mixed at various chitosan/collagen ratios of 100/0, 65/35, 25/75, or 0/100 wt%. Pre-cooled 58 wt% β-GP (Sigma-Aldrich, United Kingdom) solution was added to the above chitosan/collagen solutions to obtain a final β-GP concentration of 8%. All procedures were conducted on ice to maintain a liquid state before initiating gelation at 37°C. Gelation was determined by the mobility of chitosan-collagen solutions after inverting a test tube and the transition from clear to opaque transparency. The pH of the hydrogels was recorded before and after gelation.

### 2.2 Mechanical Testing

The Young’s modulus of the composite gels was assessed via cyclic testing using the Instron 5542 mechanical tester (Instron, United States). Cylindrical hydrogels (Ø8 mm × 2 mm) were prepared as previously described and incubated in Phosphate buffered saline (PBS, Lonza, United Kingdom) for 4 h prior to testing. Compression testing was performed at a rate of 1 mm/min to a maximum strain of 60% of the original height by performing 8 cycles of loading/unloading. The load (N) and compressive strain (mm) was assessed using the Bluehill 3 software. The Young’s modulus was calculated from the slope of the linear region of the stress (kPa)/strain (mm/mm) curves from the 8th cycle. Samples were tested in triplicate for each condition.

### 2.3 Cell Culture and Reagents

MC3T3 pre-osteoblasts were acquired from American Type Culture Collection (ATCC, United Kingdom) and hBMSCs were purchased from Lonza (Lonza, United Kingdom). Cells were cultured in basal media consisting of minimal essential medium (α-MEM; Sigma-Aldrich, United Kingdom) supplemented with 10% foetal bovine serum (FBS), 1% penicillin/streptomycin (Sigma-Aldrich, United Kingdom), and L-glutamine (Sigma-Aldrich, United Kingdom). hBMSCs were used at passage 4. Osteogenic medium was comprised of basal media supplemented with 50 μg/ml L-ascorbic acid (Sigma-Aldrich, United Kingdom) and 10 mM β-GP (Sigma-Aldrich, United Kingdom). The medium used for EV isolation and the culture of EV-functionalised hydrogels was depleted of FBS-derived EVs via ultracentrifugation at 120,000 g for 16 h prior to use.

### 2.4 The Biological Efficacy of Chitosan-Collagen Composite Hydrogel

The influence of hydrogel composition on proliferation was assessed via quantification of DNA content. Briefly, MC3T3s were mixed at low density (5 × 10^5^ cell/ml) in the hydrogel prior to gelation. Following sol-gel transition, cell-laden hydrogels were cultured in basal medium for 2 weeks with media changes every 3 days. The cellular morphology was assessed at day 3 by incubated cell-laden hydrogels with Calcein-AM (1 μg/ml in PBS, Sigma-Aldrich, United Kingdom) and Propidium iodide (1 μg/ml in PBS, Sigma-Aldrich, United Kingdom) in the dark for 30 min. Samples were observed under an EVOS fluorescent inverted microscope (Thermo Scientific, United Kingdom). The DNA content was assessed using PicoGreen (Life Technologies, United Kingdom) according to the manufacturer’s protocol.

To evaluate the hydrogel effect on osteoinduction, MC3T3s were mixed at high density (1 × 10^6^ cell/ml) in the hydrogel prior to gelation. Following sol-gel transition, hydrogels were incubated in basal medium for 24 h. The media was replaced with osteogenic medium and gels were cultured for 2 weeks, with media changes occurring every 3 days.

### 2.5 EV Isolation and Characterization

#### 2.5.1 EV Isolation

EVs were isolated from MC3T3s as previously reported (Man et al., 2021b). Briefly, osteoblasts were cultured at scale in T175 flasks in osteogenic medium for 14 days. The conditioned media was collected every two days. EVs were isolated from the conditioned media by differential centrifugation: 2000 g for 20 min, 10,000 g for 30 min, and 120,000 g for 70 min to pellet EVs. The supernatant was removed, and the pellet was washed in sterile PBS and centrifuged at 120,000 g for 70 min and the resultant pellet was re-suspended in 500 μL PBS. All ultracentrifugation steps were performed utilising the Sorvall WX Ultra Series Ultracentrifuge (Thermo Scientific, United Kingdom) and a Fiberlite, F50L-8×39 fixed angle rotor (Piramoon Technologies Inc., United States).

#### 2.5.2 Particle Size and Concentration Analysis

Nanoparticle tracking analysis was performed on isolated EVs to determine particle concentration using a ZetaView^®^ instrument (Particle Metrix, Germany). EV samples were diluted at 1:100 in PBS and injected into the ZetaView^®^, where 4 × 40 s videos were obtained of particles in motion. Particle size and concentration were determined with the ZetaView^®^ software. Dynamic Light Scattering (DLS) (Zetasizer Nano ZS, Malvern Instruments, United Kingdom) was used to analyse the size distribution of EVs before and after incorporation within the different hydrogels.

#### 2.5.3 Transmission Electron Microscopy

The morphology of isolated EVs was conducted via a JEOL JEM1400 transmission electron microscope (TEM) coupled with an AMT XR80 digital acquisition system. Samples were physiosorbed to 200 mesh carbon‐coated copper formvar grids (Agar Scientific, United Kingdom) and negatively stained with 1% uranyl acetate.

#### 2.5.4 EV Marker Analysis

The presence of tetraspanin markers on the surface of EVs was assessed using the ExoView™ Tetraspanin Kit (NanoView Biosciences, United States) according to the manufacturers’ protocol and as previously described (Gupta et al., 2020; Luo et al., 2021). Briefly, 35 µL of EV suspension (1:1,000 dilution in Incubation Solution) was incubated on the ExoView™ chip for 16 h. The chip was washed with Incubation Solution for 3 min, three times using Wash Solution and then with deionized water. The chip was then dried and analysed using the ExoView R100 (NanoView Biosciences, United States) with the nScan software (version 2.8.10). Using single particle interferometric reflectance imaging sensing (SP-IRIS), CD9 and/or CD81 tetraspanin-positive nanoparticles were detected and counted spot by spot as they were immuno-captured on the chip. Rat IgG spots were used as an isotype control. The data were analysed using the NanoViewer software (version 2.8.10) with sizing thresholds set to 50–200 nm diameter.

### 2.6 EV Release Kinetics From Hydrogels

The *in vitro* release kinetics of EVs within the chitosan-collagen hydrogels was assessed as previously reported (Nikravesh et al., 2019). Briefly, EVs were introduced into the different hydrogel formulations at the concentration of 100 µg/ml of EV protein prior to gelation. EV-functionalised gels were then incubated in sterile PBS at 37°C. At day 1, 3, 5, and 7, the receiving medium was collected, and replaced by an equal volume of fresh PBS. Previous studies have shown that EVs derived from mineralising osteoblasts were CD63 positive and as such we selected this marker for the release study (Man et al., 2021b; Man et al., 2022). The EV concentration in the collected medium was evaluated using the CD63 ExoELISA-ULTRA complete kit (System Biosciences, United States) following the manufacturer’s protocol. Percentage of EVs released was calculated from the initial quantity of EVs added prior to gelation.

### 2.7 The Impact of Hydrogel-Released EVs on hBMSCs Proliferation, Migration, and Mineralisation

#### 2.7.1 EV Cell Uptake

EVs were labelled using Cell Mask™ Deep Red Plasma Membrane Stain, 1:1,000 in PBS (Thermo Scientific, United Kingdom), and incubated for 10 min. Labelled EVs were washed twice with PBS *via* ultracentrifugation at 120,000 g for 70 min, then incorporated within the 100/0 hydrogel before gelation. hBMSCs were seeded at 3 × 10^3^ cells/cm^2^ in a 48 well plate for 24 h, then media was replaced with fresh basal medium and transwell inserts (0.4 µm pore size, Greiner Bio-One, United Kingdom) containing Cell Mask-labelled EVs encapsulated within the hydrogel. Cells cultured with EV-free hydrogels were used as the control. After 24 h, cells were fixed with 10% (v/v) neutral buffered formalin (NBF, Cellpath, United Kingdom), stained with Alexa Fluor 488 phalloidin, 1:20 (Cell Signalling Technology, United Kingdom), and mounted with Prolong™ Gold Antifade Mountant with DAPI (Thermo Scientific, United Kingdom) to label the actin cytoskeleton and nuclei, respectively. Samples were imaged with an EVOS fluorescent inverted microscope (M5000, Thermo Scientific, United Kingdom).

#### 2.7.2 Proliferation

hBMSCs were plated at low density (1 × 10^4^ cells/cm^2^) in basal medium within a 48 well plate. After 24 h, media was replaced with fresh basal medium and transwell inserts (0.4 µm pore size, Greiner Bio-One, United Kingdom), containing EV-functionalised hydrogels, were placed into each well. Media was replaced every 3 days. DNA content was assessed using the PicoGreen (Life Technologies, United Kingdom) according to the manufacturer’s protocol. Cells cultured without EV-functionalised hydrogels were used at the control. An EV only group was not included, as we focused on assessing the biological potency of EVs release from these hydrogel systems on recipient hBMSCs behaviour.

#### 2.7.3 Migration

The migration area was calculated by performing the wound healing assay. Briefly, cells at a density of 30 × 10^3^ cells/cm^2^ in a 48 well plate were plated and allowed to adhere for 24 h. A scratch was applied with a 200 µL pipette tip and the width was measured as the baseline. Cells were incubated with transwell inserts (0.4 µm pore size, Greiner Bio-One, United Kingdom) containing EV-functionalised hydrogels for 3 days. Cells cultured without hydrogels were used as the control. The area of wound closure from day 0 was assessed using fluorescent microscopy. Briefly, cells were labelled with Calcein-AM (1 μg/ml in PBS, Sigma-Aldrich, United Kingdom) in the dark for 30 min. Samples were observed under an EVOS fluorescent inverted microscope (Thermo Scientific, United Kingdom).

#### 2.7.4 hBMSCs Osteoinduction

hBMSCs were seeded at high density (3 × 10^3^ cells/cm^2^) in basal medium within a 48 well plate. After 24 h, media was replaced with osteogenic medium and transwell inserts (0.4 µm pore size, Greiner Bio-One, United Kingdom) containing EV-functionalised hydrogels were placed in each well. Cells cultured without hydrogels were used as the control. Media was replaced every 3 days.

### 2.8 EV-Functionalised Hydrogels on hBMSCs Proliferation and Mineralisation

The viability and proliferation of hBMSCs (5 × 10^5^ cell/ml) within the EV-functionalised 65/35 composite hydrogels was assessed *via* live/dead staining. Briefly, hBMSCs (5 × 10^5^ cell/ml) were mixed with the EV-functionalised hydrogel (0, 50, or 100 µg/ml of EV protein) prior to gelation. Following sol-gel transition, hydrogels were culture in basal medium. At day 7, cell-laden hydrogels were incubated with Calcein-AM (1 μg/ml in PBS, Sigma-Aldrich, United Kingdom), and Propidium iodide (1 μg/ml in PBS, Sigma-Aldrich, United Kingdom) in the dark for 30 min. Samples were observed under an EVOS fluorescent inverted microscope (Thermo Scientific, United Kingdom). The proliferation of cell-laden EV-hydrogels was assessed *via* quantifying DNA content following culture in basal medium for 7 days.

The capacity of EV-functionalised hydrogels to stimulate encapsulated hBMSCs (1 × 10^6^ cell/ml) osteogenic differentiation and mineralisation was evaluated after culture in osteogenic medium for 3 weeks. Osteogenic differentiation was assessed by quantifying alkaline phosphatase activity, collagen production and mineral deposition, detailed below. Cell-free hydrogels of each composition were cultured in the same conditions as described above and used as an acellular control for the following analysis.

### 2.9 Alkaline Phosphatase Activity

ALP activity was determined using the 4-nitrophenyl colourimetric phosphate liquid assay (pNPP, Sigma-Aldrich, United Kingdom) as previously reported (Man et al., 2021a). Briefly, cell lysate was isolated from cell-laden hydrogels by five freeze/thaw cycles between −80 and 37°C, with homogenisation *via* passing through syringe/needles and sonication in between. 10 μL of cell lysate was added to 90 μL of pNPP and incubated for 60 min at 37°C. The absorbance at 405 nm was read on a SPARK spectrophotometer (TECAN, CH). ALP activity was normalised with DNA content.

### 2.10 Collagen Production

Extracellular matrix collagen deposition was evaluated with picrosirius red staining. Briefly, samples were washed twice in PBS and fixed in 10% NBF for 30 min, prior to staining with Picro-Sirius Red Solution (ScyTek Laboratories, Inc., United States) for 1 h. The unbound dye was removed by washing in 0.5 M acetic acid followed by distilled water wash and left to air dry prior to imaging using light microscopy (EVOS XL Core, Invitrogen, United Kingdom). To quantify collagen staining, 0.5 M sodium hydroxide was used to elute the bound dye and absorbance were read at 590 nm using the SPARK spectrophotometer (TECAN, CH).

### 2.11 Mineral Deposition

To investigate mineralisation, calcium deposition was assessed *via* alizarin red staining. Cells were washed twice in PBS and fixed in 10% NBF for 30 min. Following fixation, samples were washed in distilled water and then incubated with alizarin red solution (Sigma-Aldrich, United Kingdom) for 10 min. The unbound dye was removed by washing in distilled water. Staining was visualised using light microscopy (EVOS XL Core, Invitrogen, United Kingdom). For quantification, samples were eluted with 10% cetylpyridinium chloride (Sigma-Aldrich, United Kingdom) for 1 h and then absorbance was read at 550 nm using the SPARK spectrophotometer (TECAN, CH).

### 2.12 Statistical Analysis

For all data presented, experiments were performed in triplicate. All statistical analysis was undertaken using ANOVA multiple comparisons test with Tukey modification using IBM SPSS software (IBM Analytics, version 21). *p* values equal to or lower than 0.05 was considered as significant. *p ≤ 0.05, **p ≤ 0.01, ***p ≤ 0.001. All experiments were repeated independently at least three times.

## 3 Results

### 3.1 The Influence of Chitosan-Collagen Formulation on Physiochemical Properties

**FIGURE 2:**
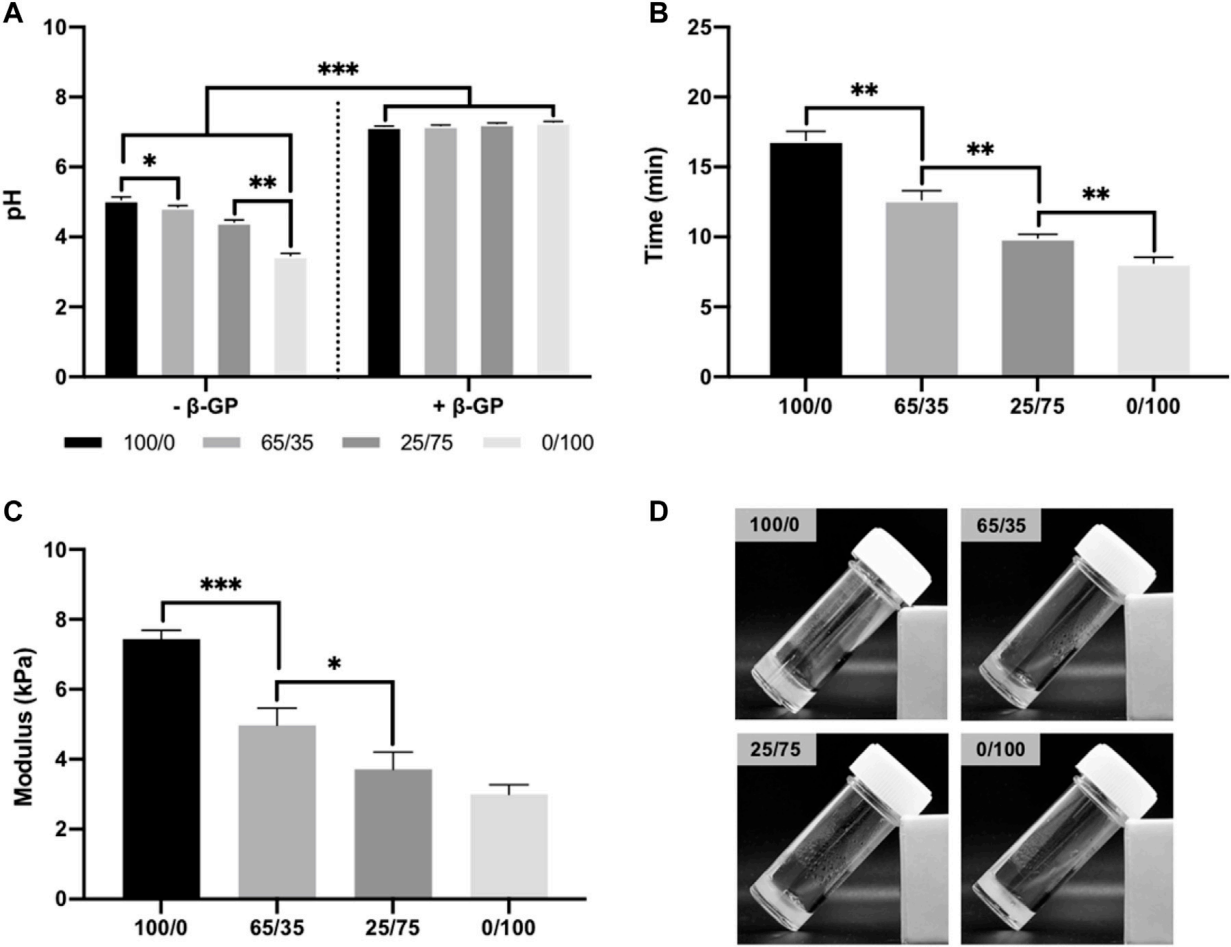
Physiochemical properties of chitosan-collagen hydrogels. **(A)** pH of composite hydrogels before and after β-GP addition. **(B)** Gelation time of hydrogels at 37°C. **(C)** Compressive modulus. **(D)** Macroscopic image of hydrogels after gelation. Data are expressed as mean ± SD (*n* = 3). *p ≤ 0.05, **p ≤ 0.01, and ***p ≤ 0.001.

Figure 2A shows the gelation parameters for the chitosan-collagen hydrogels. Pure and composite hydrogels without β-GP exhibited pH values ranging from 3.5 to 5, with higher pH values at greater chitosan concentrations (*p* ≤ 0.05–0.01). Following β-GP addition, pH for all groups were significantly elevated to approximately pH 7.3 (*p* ≤ 0.001). The gelation time of hydrogels was assessed at 37°C. Hydrogels containing increased collagen content exhibited significantly reduced gelation times when compared to chitosan-containing gels (16.9 ± 0.25 min (100/0), 12.65 ± 0.66 (65/35), 9.93 ± 0.26 (25/75), and 8.13± 0.41 min (0/100) (*p* ≤ 0.01) (Figure 2B). The compressive modulus of chitosan-collagen hydrogels is shown in Figure 2C. There was a chitosan-dependent increase in hydrogel stiffness, with compressive moduli of 7.43 ± 0.25 (100/0), 4.97 ± 0.49 (65/35), 3.71 ± 0.46 (25/75), and 2.99 ± 0.28 kPa (0/100) (*p* ≤ 0.05–0.001). Macroscopic images of chitosan-collagen hydrogels following sol-gel transition are shown in Figure 2D, where the gels have transitioned from a clear to opaque transparency and displayed no mobility during inversion testing.

### 3.2 The Effects of Chitosan-Collagen Hydrogel Composition on Osteogenic Differentiation.

**FIGURE 3:**
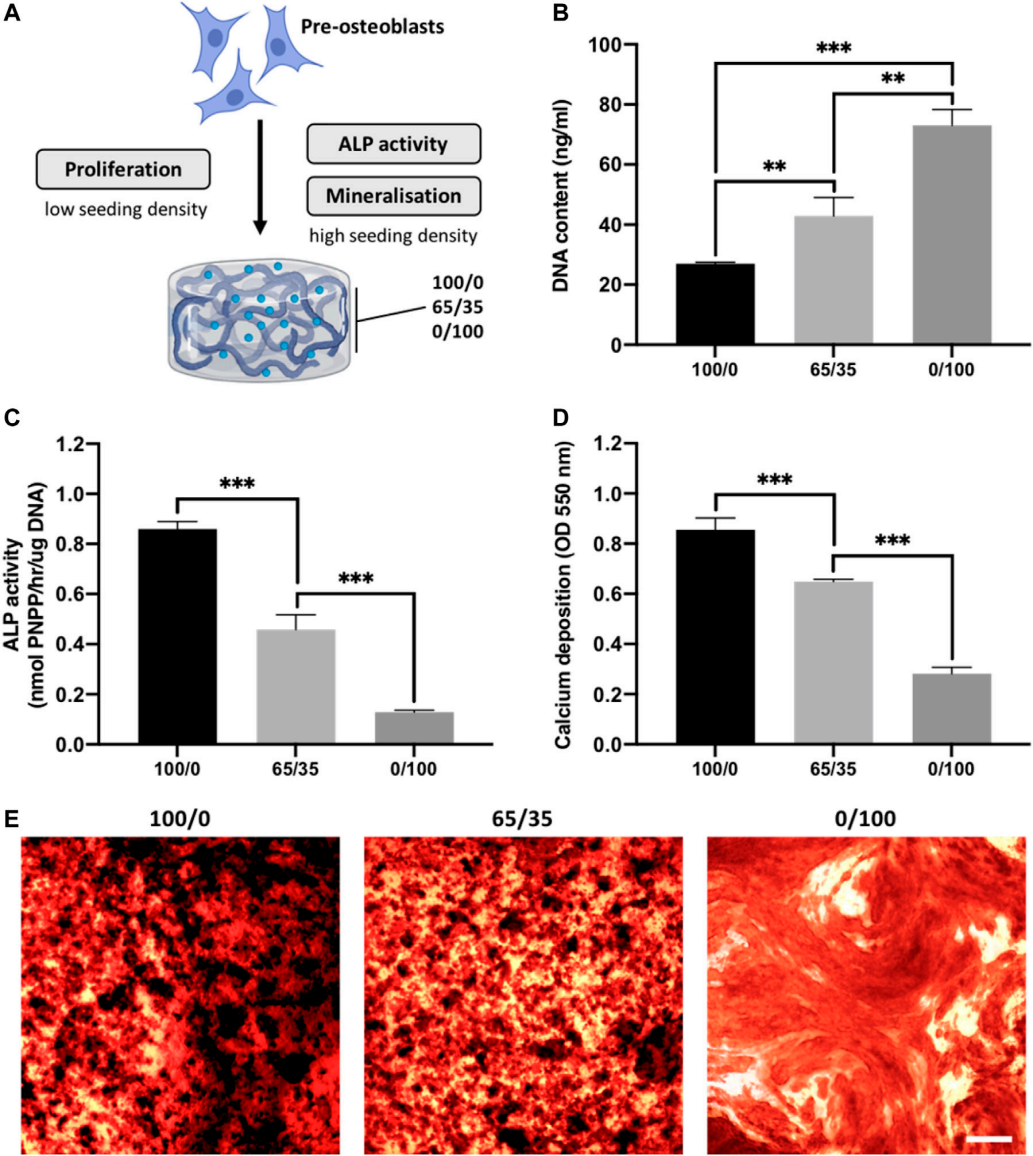
The effects of chitosan-collagen composite hydrogel on proliferation and osteogenic differentiation. **(A)** Schematic representation of functional assessments. The influence of different hydrogel formulations on **(B)** proliferation, **(C)** ALP activity and **(D,E)** calcium deposition. Black staining indicates mineral nodules. Scale bar = 200 µm. Data are expressed as mean ± SD (*n* = 3). *p ≤ 0.05, **p ≤ 0.01, and ***p ≤ 0.001.

The biological effects of altering chitosan-collagen content within the composite hydrogel on cell behaviour was evaluated (Figure 3A). The morphology of encapsulated cells within the different hydrogel compositions were initially assessed. At day 3 in culture, cells in the 0/100 gels exhibited a rounded morphology, while the encapsulated cells in the collagen-containing gels displayed a spindle-shaped morphology (Supplementary Figure S1). Moreover, these images indicated cell proliferation was increased in the collagen-containing gels, which was then evaluated through DNA quantification. Hydrogels containing increased collagen proportions exhibited enhanced DNA content, where the 0/100 gels displayed a 1.70-fold (*p* ≤ 0.01), and 2.71-fold (*p* ≤ 0.001) significant increase compared to the 65/35 and 100/0 groups respectively after 2 weeks of culture (Figure 3B). The osteogenic capacity of chitosan-collagen composites was initially assessed by quantifying ALP activity. Cells cultured within hydrogels containing more chitosan displayed enhanced ALP activity, with the 100/0 group exhibiting a 1.87 (*p* ≤ 0.001) and 6.61-fold (*p* ≤ 0.001) significant increase compared to the 65/35 and 0/100 gels respectively (Figure 3C). A similar profile on calcium deposition was observed, with the cells cultured within the 100/0 gels exhibiting a 1.32 (*p* ≤ 0.001) and 3.07-fold (*p* ≤ 0.001) enhancement in calcium content when compared to the 65/35 and 0/100 groups (Figures 3D, E). An increased density of mineral-like nodules (black staining) were observed in the gels containing increased chitosan.

### 3.3 Characterisation of Isolated EVs and Hydrogel Release Kinetics

**FIGURE 4:**
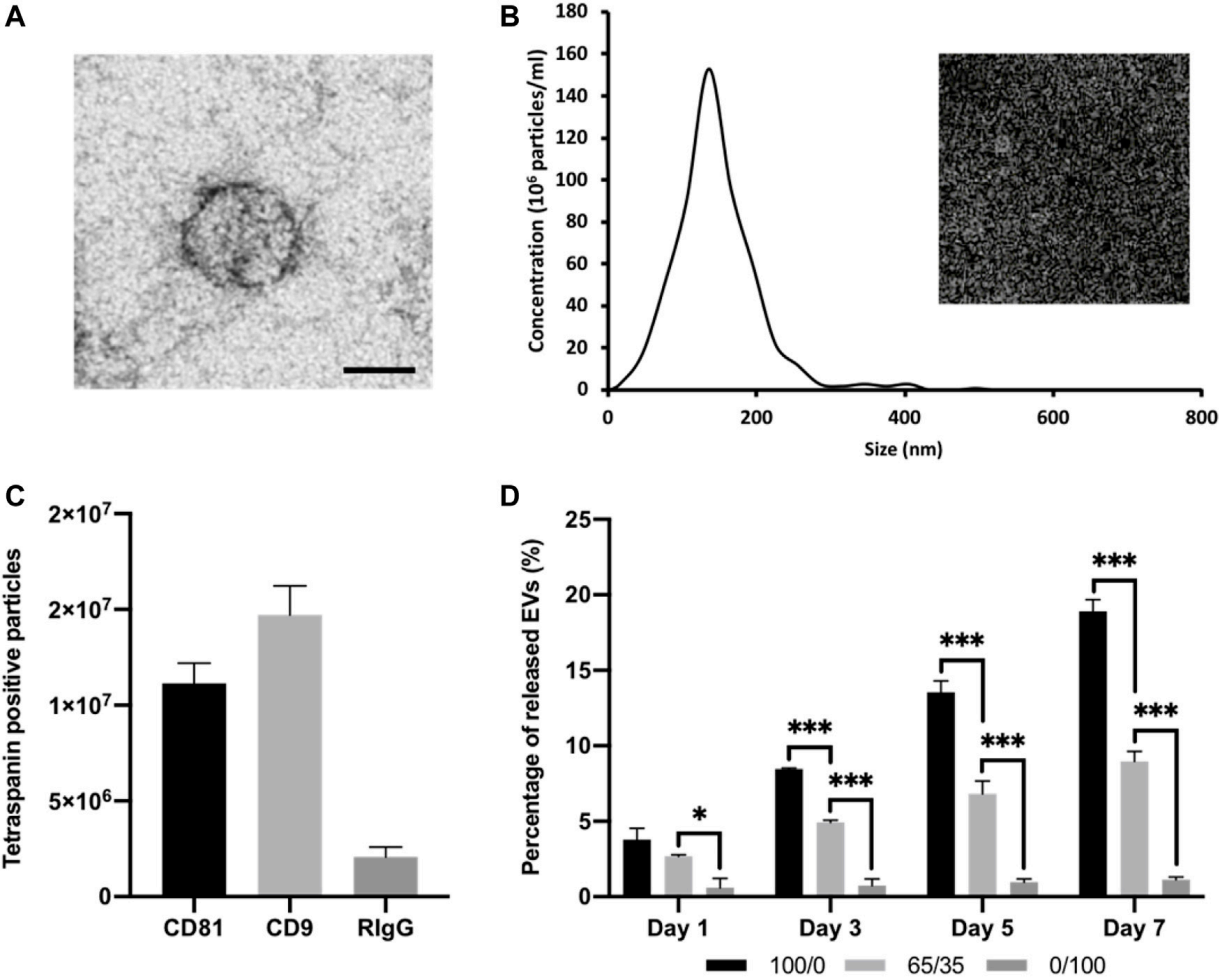
Characterisation of isolated osteoblast-derived EVs and hydrogel-EV release kinetics. **(A)** TEM image of EVs. Scale bar = 50 nm. **(B)** Size distribution of isolated EV by NTA. Insert shows snapshot of particles during analysis. **(C)** Detection of tetraspanin-positive nanoparticles (CD81 and/or CD9) *via* interferometry after immuno-capture onto ExoView™ chip. **(D)** Quantification of EVs released from chitosan-collagen hydrogels assessed via CD63 positive ELISA. Data are expressed as mean ± SD (*n* = 3). *p ≤ 0.05, **p ≤ 0.01, and ***p ≤ 0.001.

EV were isolated from mineralising osteoblast conditioned media over a 2-week period via differential centrifugation. Osteoblast-derived EVs exhibited a typical size and spherical morphology through TEM imaging (Figure 4A). NTA analysis of isolated EVs displayed an average diameter of 131.3 ± 11.4 nm (Figure 4B). The detection of EV tetraspanin markers was performed using the Exoview platform. The obtained data confirmed the presence of CD9 and CD81-positive nanosized particles immune-captured on anti-CD81 and CD9 antibodies (Figure 4C). The EV release kinetics from these composite hydrogels were investigated using the CD63 ELISA (Figure 4D). The release of EVs from these hydrogel formulations was dependant on chitosan/collagen ratios, with an increased quantity of CD63 positive particles released from gels containing a greater proportion of chitosan. There was a high average correlation between the release of EVs and the proportion of collagen within the hydrogel (R^2^ > 0.94; Supplementary Figure S2). The pure chitosan formulations (100/0) released a significantly enhanced quantity of CD63 positive particles when compared to the hydrogels containing collagen (65/35 and 0/100) at day 1, 3, 5, and 7 (*p* ≤ 0.001). At day 1, 3.78 ± 0.76% (100/0), 2.68 ± 0.09% (65/35), and 0.6 ± 0.61% of CD63 positive EVs were released. At day 7, the quantity of CD63 positive EVs released from the gels increased to 18.91 ± 0.77% (100/0), 8.98 ± 0.66% (65/35), and 1.13 ± 0.18% (0/100). The size distribution of EVs before and after hydrogel incorporation did not significantly shift over time between the different hydrogel groups (Supplementary Figure S3).

### 3.4 Hydrogel-Released EVs Enhance hBMSC Proliferation and Osteogenic Differentiation

**FIGURE 5:**
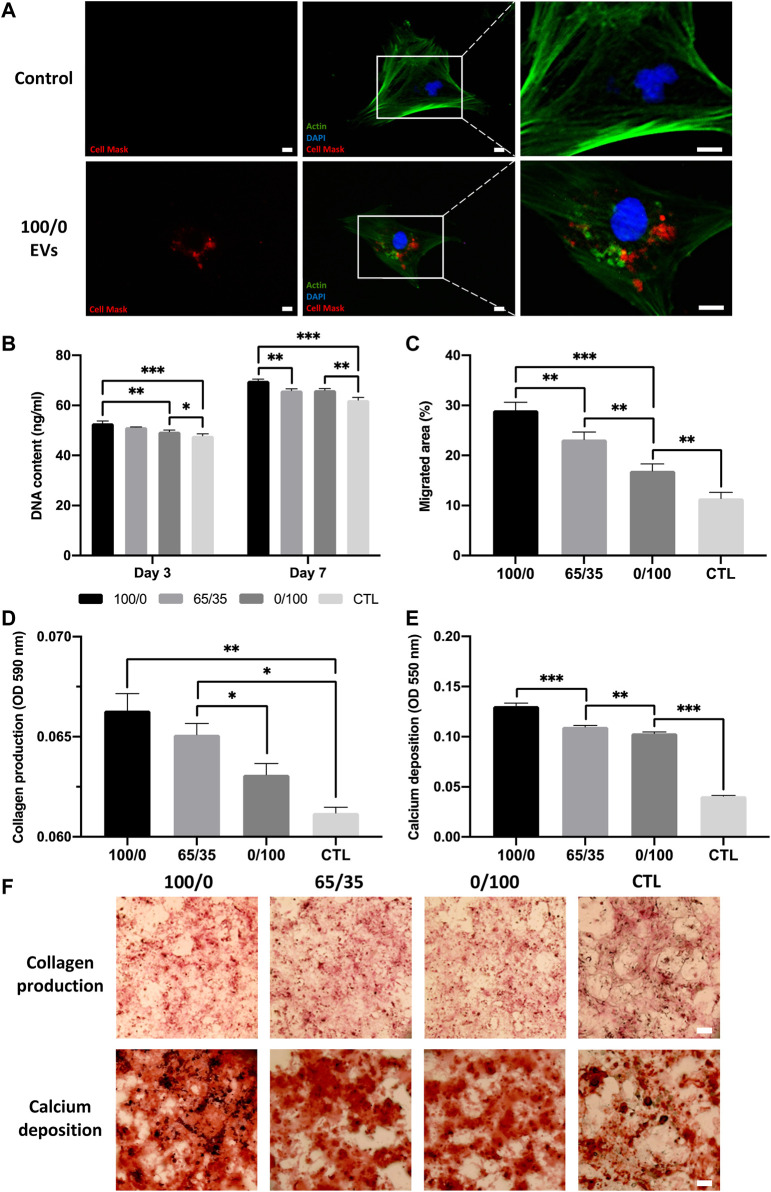
The biological efficacy of hydrogel-released EVs on hBMSCs behaviour. The influence on hydrogel-released EVs on hBMSCs **(A)** EV cell uptake. Scale bar = 20 μm, **(B)** proliferation, **(C)** migration, **(D,F)** collagen production, and **(E,F)** calcium deposition. Black staining indicates mineral nodules. Scale bar = 100 µm. Data are expressed as mean ± SD (*n* = 3). *p ≤ 0.05, **p ≤ 0.01, and ***p ≤ 0.001.

The therapeutic efficacy of hydrogel-released EVs on hBMSCs behaviour was evaluated using the transwell assay. We observed the successful internalisation of hydrogel-released EVs by hBMSCs, with the labelled vesicles primarily located within the cytoplasm of the cell (Figure 5A). There was a significant increase in the proliferation of hBMSCs in all EV-functionalised hydrogel groups when compared to the untreated control (*p* ≤ 0.001) (Figure 5B). Notably, there was a chitosan-dependent increased in hBMSCs proliferation at day 3 and 7. Cells cultured with the 100/0 group exhibited a significantly enhanced DNA content when compared to the 0/100 (1.07-fold, *p* ≤ 0.01) and untreated control (1.10-fold, *p* ≤ 0.001) at day 3. At day 7, a similar trend was observed, where the 100/0 group showed a significantly enhanced hBMSCs DNA content compared to the cells cultured with the 65/35 (1.06-fold, *p* ≤ 0.01) and 0/100 (1.06-fold, *p* ≤ 0.01) and untreated control (1.12-fold, *p* ≤ 0.001). The EV functionalised hydrogels significantly promoted hBMSCs migration when compared to the untreated control (>1.48-fold, *p* ≤ 0.01–0.001) (Figure 5C) (Supplementary Figure S4). A chitosan-dependent increase in migration was observed, where the 100/0 group exhibited a 1.25 (*p* ≤ 0.01) and 1.72-fold (*p* ≤ 0.001) enhancement in hBMSCs migration compared to 65/35 and 0/100, respectively.

The effects of hydrogel-released EVs on hBMSCs osteogenic differentiation was evaluated by analysing collagen production and calcium deposition during osteogenic differentiation. We observed a significant increase in osteogenic differentiation in all EV-functionalised hydrogel groups when compared to the untreated control following 2 weeks of osteoinductive culture (*p* ≤ 0.05–0.001). There was a chitosan-dependent enhancement in hBMSCs collagen production, with the 100/0 group exhibited a 1.02 (*p* > 0.05), 1.05 (*p* ≤ 0.05), and 1.08-fold (*p* ≤ 0.01) greater collagen content compared to 65/35, 0/100 and untreated control respectively (Figures 5D, F). The effect on calcium deposition showed a similar profile, with the pure chitosan groups exhibiting a significant 1.19, 1.26, and 3.25-fold increase in hBMSCs calcium accumulation compared to the 65/35, 0/100, and untreated group respectively (*p* ≤ 0.001) (Figures 5E, F).

### 3.5 EV-Functionalised Chitosan-Collagen Composite Hydrogel Promoted hBMSC Osteogenic Differentiation and Mineralisation

**FIGURE 6:**
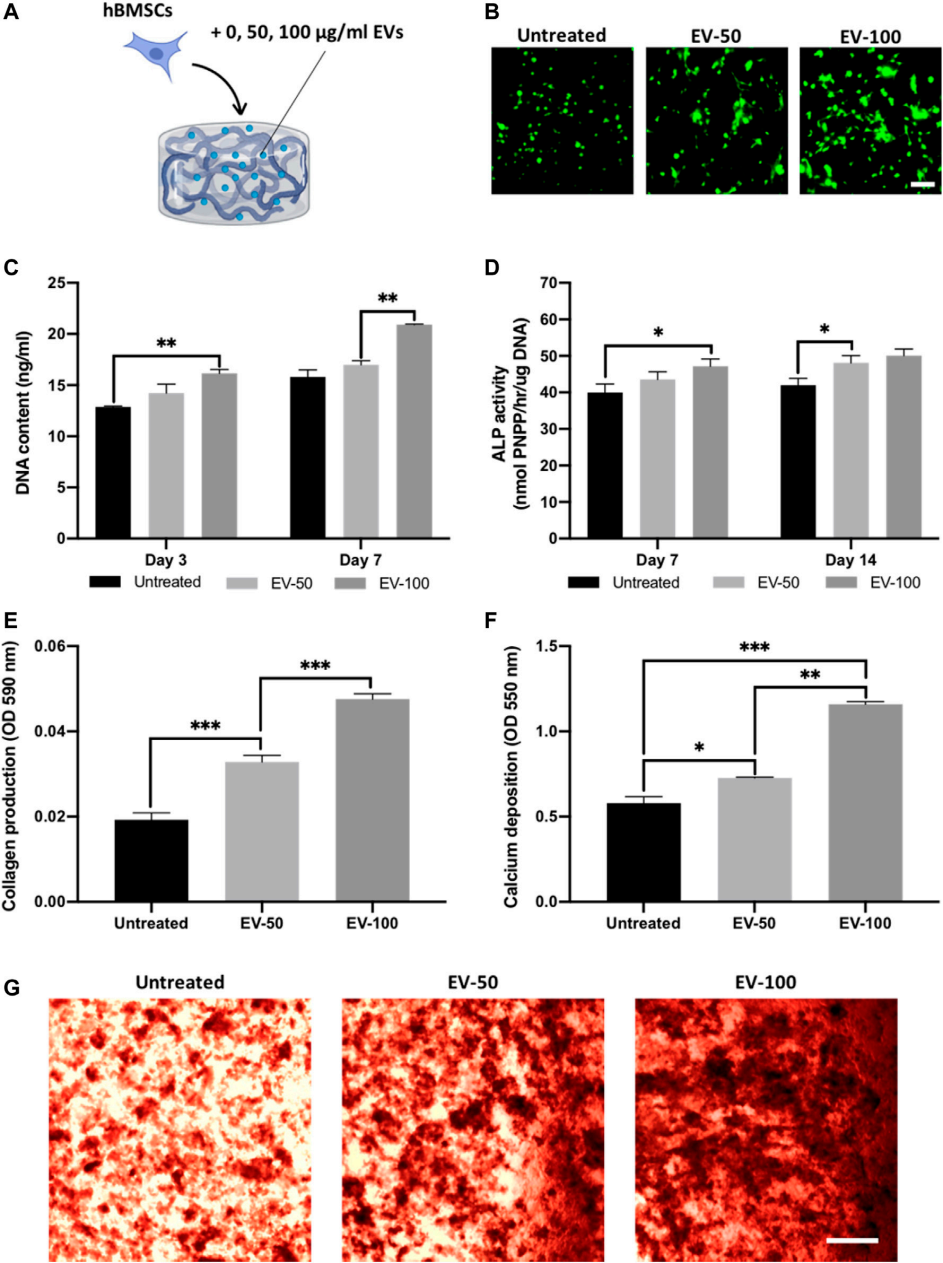
The effect of EV-functionalised chitosan-collagen hydrogel on encapsulated hBMSCs osteogenic differentiation. **(A)** Schematic representation of EV-hydrogel groups (0 µg/ml = untreated, 50 µg/ml = EV-50, 100 µg/ml = EV-100). **(B)** Live/dead staining of encapsulated hBMSCs after 7 days of culture. Scale bar = 200 µm. **(C)** DNA content within EV-hydrogels after 7 days of culture. The influence on EV-hydrogels on hBMSCs **(D)** ALP activity, **(E)** collagen production, and **(F,G)** calcium deposition during osteogenic culture. Black staining indicates mineral nodules. Scale bar = 200 µm. Data are expressed as mean ± SD (*n* = 3). *p ≤ 0.05, **p ≤ 0.01, and ***p ≤ 0.001.

The 65/35 composite was utilised as it provides a suitable balance between compressive modulus, osteogenic properties and EV release kinetics. Hydrogels were functionalised with 0, 50, or 100 ug/ml of EV protein (Figure 6A), and the viability of hBMSCs was assessed with live/dead staining and DNA quantification. At day 7, live/dead imaging showed an EV dose-dependent increase in the density of viable cells when compared to the untreated control (Figure 6B). There was a significant enhancement in hBMSC proliferation in an EV dose-dependent manner at day 3 (untreated *vs.* EV-100, *p* ≤ 0.01) and 7 (EV-50 *vs.* EV-100, *p* ≤ 0.01) (Figure 6C).

The influence of EV-functionalised hydrogels on the mineralisation of encapsulated hBMSCs was assessed following 21 days osteogenic culture. Quantification of ALP activity was initially conducted to evaluate the osteoinductive capacity of EV-hydrogels. An EV dose-dependent increase in ALP activity was observed from the hBMSCs at day 7 (untreated *vs.* EV-100, *p* ≤ 0.05) and day 14 (untreated *vs.* EV-50, *p* ≤ 0.01) in osteogenic culture (Figure 6D), with the EV-100 group exhibiting a non-significant increase in ALP compared to the EV-50 gels at both time points (*p* > 0.05). Extracellular matrix production and mineralisation were evaluated via quantifying collagen production and calcium deposition respectively. Both EV-50 and EV-100 groups exhibited a significant increase in collagen production when compared to the untreated control (1.68 and 2.47-fold, respectively) (*p* ≤ 0.001), with the EV-100 group eliciting a 1.46-fold enhancement compared to the EV-50 gel (*p* ≤ 0.001) (Figure 6E). A similar trend was observed for calcium deposition, with the EV-functionalised gels displaying significantly enhanced calcium content compared to the untreated control. The EV-50 and EV-100 gels exhibited a 1.28-fold (*p* ≤ 0.05) and 2.03-fold (*p* ≤ 0.001) increased in calcium deposition when compared to the untreated control, with the EV-100 gel displaying a significantly greater degree of calcium deposition and mineral-like nodules when compared to the EV-50 group (1.58-fold) (*p* ≤ 0.01) (Figures 6F, G).

## 4 Discussion

Harnessing EVs as an acellular tool for bone augmentation strategies has gained considerable interest in recent years (Marolt Presen et al., 2019; Man et al., 2021b). For example, Davies *et al.* demonstrated that osteoblast-derived EVs enhanced hBMSCs mineralisation when compared to the use of the clinically relevant growth factor BMP2 (Davies et al., 2017). Due to the rapid clearance of systemically administered vesicles from the body (Murphy et al., 2019; Elsharkasy et al., 2020), the logical next step to advance EV-based therapies to the clinical setting, would be the development of an osteoinductive delivery system to enhance EVs bioavailability and bioactivity to promote bone repair. As cell-derived factors such as EVs are known to interact with ECM components during mineralisation, the delivery of vesicles within an ECM-mimetic biomaterial could recapitulate the EV function during bone formation. Therefore, in this study, we investigated the development of a pro-osteogenic chitosan-collagen composite hydrogel to control EV release kinetics and synergistically promote EV-induced hBMSCs osteogenesis.

In recent years, a growing number of studies have investigated combining EVs with hydrogel systems to promote their release kinetics for different therapeutic applications (Riau et al., 2019; Man et al., 2020). For instance, Nikravesh *et al.* reported the development of a tailorable alginate-based EV release system, by controlling the physical structuring of the hydrogel during the sol-gel transition (Nikravesh et al., 2019). Although the delivery of these nanoparticles from hydrogels has produced promising results, the administered biomaterial must also provide appropriate physical and biological properties to support the defect site and promote *de novo* bone formation respectively. As such, we investigated the influence of altering chitosan-collagen content on the general material properties of the hydrogel. Our findings showed that the chitosan-collagen formulations exhibited gelation times ranging from 5 to 15 min at 37°C, a clinically reasonable handling time for orthopaedic surgeries (Costa, 2017). Moreover, increasing chitosan content within the hydrogel, significantly enhanced the compressive modulus of the hydrogel, consistent with studies in the literature (Chicatun et al., 2013; McBane et al., 2013). It has been reported that materials which exhibit increased stiffness, promote osteogenesis through enhanced mechanotransductive stimulation (Ghasemi-Mobarakeh et al., 2015; Sun et al., 2018), likely impacting the osteoinductive capacity of these gels. Together these findings demonstrate the composite hydrogels exhibit suitable *in situ* gelation times and mechanical properties facilitating its minimally invasive administration to the defect site.

Although several reports have demonstrated the release kinetics of EVs over multiple days from biomaterial systems (Mardpour et al., 2019; Man et al., 2020), there have been limited investigations into the role of the delivery device on EV-induced biological activity. Chitosan and type I collagen were selected due to their biocompatible nature and their osteoinductive properties (Hesse et al., 2010; Liu et al., 2020). As such, we investigated the role of altering chitosan-collagen content within the composite hydrogel to stimulate osteogenic differentiation without EVs. Our findings showed that hydrogels containing increased chitosan content promoted osteogenic differentiation, while gels with greater collagen proportions enhanced proliferation. This distinct effect on cellular proliferation and osteogenic differentiation has been similarly observed in the literature (Wang and Stegemann, 2010; Dang et al., 2017), likely due to the respective influence of each biomaterial on the composite microstructure. It has been reported that the introduction of collagen to chitosan hydrogel increases the porosity of the hydrogel (Fernandes et al., 2011). Therefore, cells within collagen containing hydrogels will likely exhibit increased proliferation due to enhanced porosity, consistent with our findings. Moreover, the increased compressive modulus of chitosan containing hydrogels is expected to have a positive influence on osteogenesis due to eliciting increased mechanical stimulation (Ghasemi-Mobarakeh et al., 2015; Sun et al., 2018). As EVs are essentially fingerprints of their parental cells (Kobayashi et al., 2015), the chitosan and collagen substrates may differentially influence the osteoinductive efficacy of hydrogel-encapsulated cell secreted EVs. We previously demonstrated that 3D printed titanium scaffolds exhibiting a triangular pore conformation significantly promoted osteoblast differentiation when compared to square pore scaffolds (Man et al., 2021c). Importantly, the EVs derived from these triangular pore scaffolds significantly enhanced the mineralisation of hBMSCs when compared to osteoblast-derived EVs from square pore scaffolds. As chitosan containing hydrogels promoted mineralisation to a greater degree compared to collagen-containing gels in this study, it is likely the chitosan-laden cells secreted EVs promoted mineralisation within the hydrogel in an autocrine/paracrine manner, although this would require further investigation. Therefore, the distinct biological effects presented by chitosan or collagen within the hydrogel provides tunability depending on the clinical application.

Due to issues associated with the scalable/reproducible manufacture of EVs and their rapid clearance *in vivo* (Gimona et al., 2017), this further emphasises the importance of enhancing their bioavailability. There are numerous strategies to tether EVs to biomaterials, which have been extensively reviewed in the literature (Brennan et al., 2020; Man et al., 2020). In addition to selecting pro-osteoinductive materials, chitosan, and collagen were utilised due to their capacity to immobilise EVs via different mechanisms. Chitosan is a cationic polymer, which enables the formation of complexes with anionic molecules such as on the surface of EVs. Kumar *et al.* harnessed the electrostatic interactions of positively charged chitosan with the negative-charged membranes of vesicles to develop a simple and robust EV isolation method from a variety of biofluids (Kumar et al., 2021). Moreover, the amino and hydroxyl groups have been utilised to deliver numerous drugs and growth factors (Zhang et al., 2010). Collagen hydrogels have been reported to immobilise EVs via integrin-mediated interactions (Buzás et al., 2018). For example, Altei *et al.* demonstrated the integral role of EV-associated integrins with binding to collagen. Their findings showed that the introduction of a disintegrin inhibitor (Dis*B*a-01) inhibited the adhesion of EV-derived from MDA-MB-231 breast cancer cell on collagen coated tissue culture plastic (Altei et al., 2020). Hao *et al.* further demonstrated the importance of integrins in binding EVs to matrix proteins. Their findings showed that functionalising an integrin α_4_β_1_ ligand, LLP_2_A to a material surface enhanced MSC-derived EVs binding and improved vascularisation (Hao et al., 2020). Furthermore, calcium channelling Annexin proteins have been reported to play a pivotal role in matrix-mineralisation through collagen binding (von der Mark and Mollenhauer, 1997). Several studies have reported Annexin proteins are upregulated in mineralising EVs (Eichholz et al., 2020; Man et al., 2021b), with these proteins shown to be critical for the binding of proteoliposomes to collagen fibrils (Kirsch et al., 2000). Thus, the chitosan-collagen composite could provide a multi-faceted mechanism of immobilising EVs to facilitate their controlled release. To investigate EV discharge from these biomaterials, different chitosan-collagen formulations were tested in regard to their EV release kinetics over 7 days. Our findings showed that an increased proportion of chitosan within the composite enhanced the release rate of EVs from the hydrogel system. This may be due to the differential EV affinity and binding to ECM proteins. Studies have shown that the addition of β-GP to chitosan reduces the cationic nature of the hydrogel, possibly affecting its capacity to capture EVs (Kolawole et al., 2019). Our finding indicates that collagen exhibited an increased efficacy to sequester EVs within the composite material compared to chitosan. As EVs have been demonstrated to promote cell recruitment, an important process for endogenous bone repair (Wang et al., 2018), these results suggest the importance of incorporating chitosan in the composite to facilitate the release of these vesicles from the hydrogel. Together, these findings demonstrate the importance of investigating the release kinetics of EV-functionalised hydrogels, as this will significantly impact the vesicles bioavailability and functionality *in situ*.

A potential concern with the delivery of EVs within biomaterials systems, is the possibility that the released nanoparticles functionality may be altered, ultimately impacting their therapeutic efficacy. Moreover, in the bone context, the recruitment of endogenous cells into the defect site/implanted material is essential for successful *de novo* tissue formation and osseointegration (Wang et al., 2018). As EVs have been demonstrated to stimulate cellular recruitment (Gholami et al., 2021; Zhu et al., 2021), this highlights the importance of investigating the therapeutic efficacy of hydrogel-released EVs from the composite material. We reported that there were no significant changes in the size distribution of EVs before and after hydrogel incorporation and between the different formulations. This suggests EV incorporation into the material did not cause substantial damage to the nanoparticle integrity or cause vesicle aggregation. Regarding the functionality of released EVs, we initially investigated the influence of these nanoparticles on stimulating the migration and proliferation of hBMSCs, a critical process for recruitment of endogenous cells into the defect site (Perez et al., 2015). Our findings showed a chitosan-dependent increased in migration and proliferation rate induced by EV treatment, consistent with the EV release kinetics observed from these gels. This indicates that the released EVs exhibited a dose-dependent effect on hBMSCs migration and proliferation, signifying the EVs retained their biological efficacy in stimulating cellular recruitment, consistent with findings in the literature (Mol et al., 2019). In addition to the role of hydrogel-released EVs on cellular recruitment, their capacity to stimulate hBMSCs osteogenesis was investigated. EVs released from the chitosan gel significantly enhanced hBMSCs mineralisation when compared to the cells cultured with the collagen-released EVs. These findings were consistent with the influence of hydrogel-released EVs on proliferation and migration, indicating the increased quantity of EVs released from chitosan-containing gels promoted hBMSCs osteogenesis. Together, these findings indicate that the incorporated vesicles retained their biological potency once released from the hydrogel, demonstrating the viability of this composite hydrogel as a suitable delivery vehicle for these pro-osteogenic nanoparticles.

For the development of an optimal EV delivery system, it is important to consider the biomaterials role on EV release and their functionality, but also to provide a suitable environment that facilitates tissue-specific regeneration. Several studies have demonstrated the osteoinductive capacity of chitosan and collagen biomaterials (Wang and Stegemann, 2010; Sun et al., 2011), thus providing an appropriate platform to support EV-induced regeneration. Moreover, to initiate thermo-gelation in chitosan hydrogels, β-GP was incorporated to facilitate its sol-gel transition at physiological temperatures. It has been previously reported that osteoblast-derived EVs require a phosphate-rich environment to facilitate hBMSCs mineralisation (Davies et al., 2017). Therefore, the incorporation of β-GP provides the sol-gel transition of chitosan and a phosphate source to promote EV-induced mineralisation. Hence, in this present study, we investigated the influence of EV-functionalised chitosan-collagen hydrogel to stimulate encapsulated hBMSCs osteogenic differentiation. In this work, the 65/35 composite was utilised as it provides a suitable balance between compressive modulus, osteogenic properties and EV release kinetics. Moreover, several studies have reported the successful utilisation of chitosan-collagen hydrogels at similar ratios (Wang and Stegemann, 2010; Sun et al., 2011). We initially showed that the encapsulated hBMSCs remained viable within the 65/35 composite hydrogel, consistent with findings in the literature (Wang and Stegemann, 2010). Moreover, our results showed an EV dose-dependent increase in cell proliferation within the hydrogel. This suggest that the incorporated EVs retained their biological functionality, further promoting hBMSCs proliferation in a dose-dependent manner. Importantly, we demonstrated that the EV-functionalised composite gels significantly enhanced hBMSCs osteogenic differentiation and mineralisation when compared to the EV-free groups, consistent with observations in the literature (Man et al., 2021b). Similar to the effects on proliferation, our findings showed an EV dose-dependent increase in mineralisation within the hydrogel, consistent with the concentration-dependent increase in hBMSCs mineralisation observed within a GelMA nanocomposite hydrogel (Man et al., 2022). These findings further signify the efficacy of encapsulated EVs in stimulating hBMSCs osteogenesis within this hydrogel system. Taken together, these findings demonstrate the 65/35 composite hydrogel provides a biocompatible environment that facilitates EV-induced stem cell mineralisation, indicating the therapeutic viability of this acellular approach to stimulate bone regeneration.

In summary, we demonstrated the development of an injectable pro-osteogenic chitosan-collagen composite hydrogel capable of controlling the release of osteoblast-derived EVs and promote the recruitment/mineralisation of hBMSCs. We reported the impact of changing chitosan-collagen content on augmenting the hydrogels gelation time, compressive modulus, osteoinductive properties and EV release kinetics. Moreover, we showed that EVs either released or encapsulated within the composite hydrogel retained their capacity to stimulate hBMSCs proliferation and mineralisation. Having demonstrated the influence of chitosan and collagen composition on EV release kinetics *in vitro*, future *in vivo* testing will be employed to further validate the capacity of this ECM-composite hydrogel to improve EV half-life*.* As it is difficult to replicate the systemic or local delivery of EVs *in vitro*, this would be an important group to include in future *in vivo* testing*.* Moreover, future studies investigating the potential of EV-functionalised chitosan-collagen hydrogels to stimulate bone regeneration within critical-sized bone defects *in vivo* would provide increased pre-clinical evidence into the efficacy of this EV-based approach to promote bone regeneration.

## Conclusion

Together, these findings demonstrate the development of an injectable thermosensitive chitosan-collagen composite hydrogel capable of enhancing the therapeutic efficacy of osteoblast-derived EVs as novel acellular tools to promote bone regeneration.

## Data Availability

The raw data supporting the conclusion of this article will be made available by the authors, without undue reservation.

## Supplementary Material

Supplementary Figure 1 Morphology of pre-osteoblast within chitosan-collagen hydrogels following 3 days in basal culture. Scale bar = 200 µm.

Supplementary Figure 2 Pearson correlation of EV cumulative release kinetics within composite hydrogel.

Supplementary Figure 3 The size distribution of EVs before and after incorporation into hydrogel systems analysed *via* DLS analysis.

Supplementary Figure 4 The effect of hydrogel-released EVs on hBMSCs migration rate at day 3. White dash lines indicate initial baseline scratch. Scale bar = 100 µm.
