# Supplementary material for: An ECM-Mimetic Hydrogel to Promote the Therapeutic Efficacy of Osteoblast-Derived Extracellular Vesicles for Bone Regeneration
Source: Front Bioeng Biotechnol. 2022 Mar 30;10:829969. doi: 10.3389/fbioe.2022.829969 (PMC9005798; doi:10.3389/fbioe.2022.829969)
Supplement: Supplementary file 1 [file DataSheet1.docx]

**Supplementary materials**

**
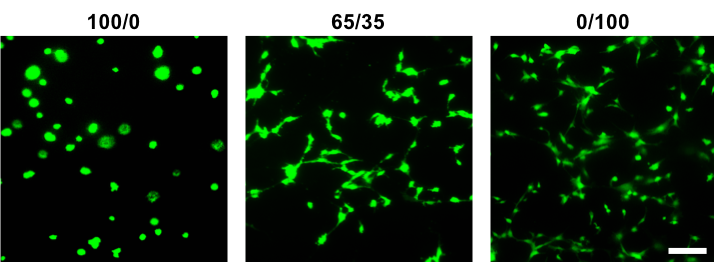
**

**Supplementary figure 1. Morphology of pre-osteoblast within chitosan-collagen hydrogels following 3 days in basal culture.** Scale bar = 200 µm.

**
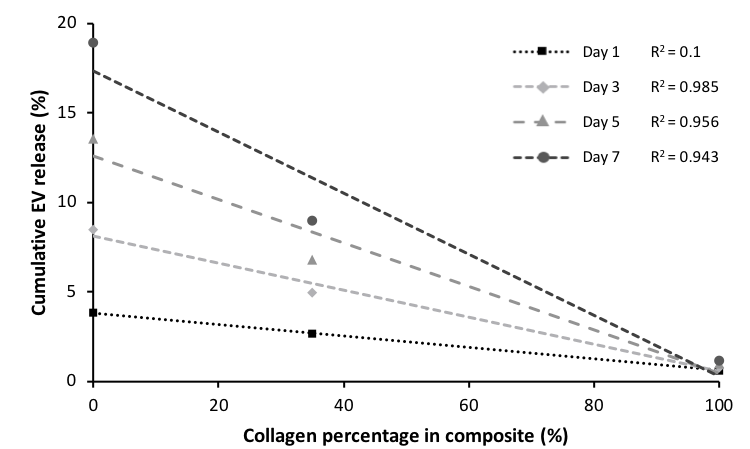
**

**Supplementary figure 2. Pearson correlation of EV cumulative release kinetics within composite hydrogel.**

**Supplementary figure 3. The size distribution of EVs before and after incorporation into hydrogel systems analysed via DLS analysis.**

**
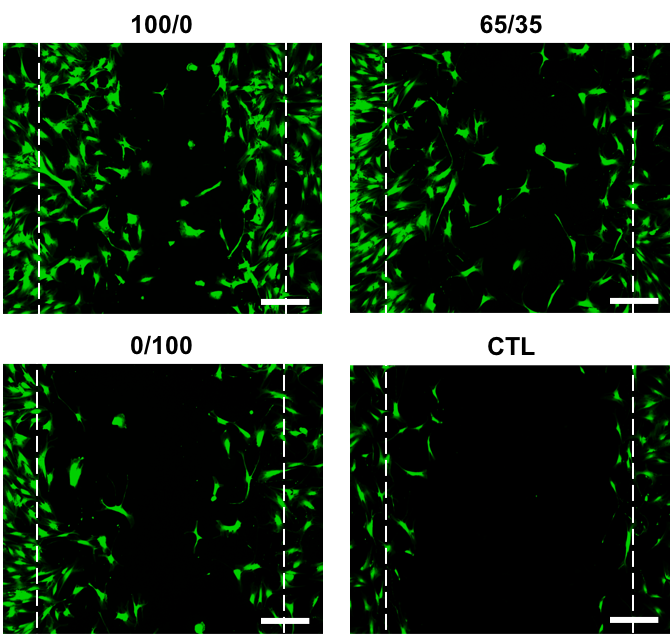
**

**Supplementary figure 4. The effect of hydrogel-released EVs on hBMSCs migration rate at day 3.** White dash lines indicate initial baseline scratch. Scale bar = 100 µm.
